# Modulation of cerebral cortex activity by acupuncture combined with continuous theta-burst stimulation in post-stroke upper limb spasticity: an fNIRS study

**DOI:** 10.3389/fneur.2025.1542489

**Published:** 2025-06-02

**Authors:** Junfeng Zhang, Meng Wang, Hao Chen, Shiyu Fan, Rongshan Sun, Lin Wang, Shan Cong, Tao Yu, Yulin Qian

**Affiliations:** ^1^Rehabilitation Center, First Teaching Hospital of Tianjin University of Traditional Chinese Medicine, Tianjin, China; ^2^National Clinical Research Center for Chinese Medicine Acupuncture and Moxibustion, Tianjin, China; ^3^Graduate School, Tianjin University of Traditional Chinese Medicine, Tianjin, China; ^4^Rehabilitation Department, Tianjin Xiqing District Hospital of Traditional Chinese Medicine, Tianjin, China

**Keywords:** continuous theta burst stimulation, acupuncture, stroke, spasticity, fNIRS, F-wave

## Abstract

**Objective:**

To observe the modulation and clinical efficacy of acupuncture combined with continuous theta-burst stimulation (cTBS) on cerebral cortical activity in post-stroke upper limb spasticity.

**Methods:**

Patients with upper limb spasticity after stroke were randomly divided into two groups. The control group (*n* = 15) received acupuncture treatment, and the experimental group (*n* = 15) received cTBS treatment of the premotor cortex of the healthy side in addition to acupuncture. The efficacy was evaluated before and after the first treatment and 2 weeks after treatment. MAS, FMA, MBI, fNIRS and F wave evaluation were observed in the two groups.

**Results:**

After treatment, there were significant differences between the two groups in MAS, FMA-UE, MBI, F-wave amplitude, and the F/M amplitude ratio index of the experimental group (*p <* 0.05). Comparison between groups, the MAS-elbow flexor in the experimental group decreased significantly after the first treatment and 2 weeks after treatment. After 2 weeks of treatment, the HbO_2_ concentration in the CH4 channel exercise phase in the experimental group increased compared with the control group (*p*_FDR_ <0.05). The strength of functional connection in the left primary motor area (M1)—the left primary somatosensory cortex (S1), the left S1—the right premotor cortex and supplementary motor area (PMC & SMA), and the left S1—the left PMC&SMA showed an increasing trend (0.05 < *p*_FDR_ < 0.1). The F wave amplitude was significantly lower than before intervention, and the difference was significant (*p* < 0.05).

**Conclusion:**

Acupuncture combined with cTBS can relieve upper limb spasticity, enhance motor function, improve daily living ability, and reduce excessive spinal cord excitability. It can also increase HbO_2_ concentration in the cerebral cortex. However, large-sample studies are still needed to prove the effect of this therapy on brain networks.

**Significance:**

This study provides evidence for the potential of combined cTBS and acupuncture in stroke rehabilitation.

**Clinical trial registration:**

identifier ChiCTR2400083631.

## 1 Introduction

Post-stroke spasticity (PSS) refers to an elevation in the muscle tone in the hemiplegic limb following a stroke. It is characterized increased tensor reflex and hyperreflexia of the tendon reflexes, typical of upper motor neuron injury (1, 2). It is estimated that PSS can affect as many as 65% of stroke survivors, hindering their recovery of motor functions and resulting in severe physical disabilities (3). A study have demonstrated an increasing incidence of PSS, particularly in the upper extremities (4). Some studies have indicated that the incidence and severity of upper limb spasticity after stroke are higher compared to those of the lower limb (5). Consequently, early intervention for upper limb spasticity following stroke is deemed highly essential (6). Furthermore, spastic paralysis imposes a significant economic burden on patients. Stroke patients with spasticity incur medical costs four times higher than those without (7). The raising prevalence and treatment costs necessitates the search for the development of innovative rehabilitation programs targeting post-stroke upper limb spasticity.

Currently recognized treatments for PSS include pharmacological interventions with botulinum toxin and non-pharmacological approaches such as stretching maneuvers. However, these treatments primarily focus on peripheral interventions. Their efficacy is often limited and non-enduring. Studies have shown that while botulinum toxin can effectively treat PSS for up to 3 months, it cannot improve voluntary motor function (8). Although traction manipulation provides immediate relief for PSS, its short- and long-term effects remain uncertain (4). The identification of safe and effective treatment protocols for PSS continues to be an urgent challenge that needs addressing.

Acupuncture is recognized by the World Health Organization (WHO) as an alternative and complementary therapy for stroke treatment and has long been utilized for the management of PSS (9). The latest systematic review indicates that acupuncture can effectively enhance the recovery rate of spasticity and reduce scores on the Modified Ashworth Scale (MAS) (10). Notably, the incidence of adverse reactions associated with acupuncture is substantially lower than that observed with traditional Western medical therapies (11). Research suggests that acupuncture promotes the alleviation of spasticity by modulating pathways within the spinal cord. Specifically, acupuncture of the antagonist muscles of spastic muscles can reduce the excitability of *α* and *γ* neurons, thereby facilitating presynaptic inhibition. Additionally, acupuncture can decrease the H_max_/M_max_ ratio and prolong the mean H-reflex recovery time, both of which contribute to the improvement of spasticity (12).

Continuous theta burst stimulation (cTBS), an innovative patterned variant of transcranial magnetic stimulation (TMS) (13), stands alongside high-frequency repetitive TMS (rTMS) and low-frequency rTMS as a powerful paradigm in neuromodulation (14, 15). Recent clinical investigations have underscored its efficacy in ameliorating upper limb spasticity symptoms in stroke patients when administered to the unaffected hemisphere of the brain. Notably, cTBS exhibits sustained superiority over low-frequency rTMS in this regard, demonstrating persistent therapeutic benefits (16). Since nerve fibers originating from the premotor area (PMA) of the brain play a pivotal role in regulating limb muscle tone, the unaffected PMA was targeted as the stimulation site (17).

Presently, the intricate pathogenesis of post-stroke upper limb spasticity remains enigmatic, with a multitude of studies underscoring its association with the dysfunctional imbalance of the reticulospinal tract (RST) (18, 19). However, some studies have also proposed that the occurrence of PSS is inextricably linked to the imbalance between excitation and inhibition within the cortex (3) or spinal cord (20). Functional near-infrared spectroscopy (fNIRS), an emerging brain imaging technique, represents a potent tool for assessing the functional status of the cerebral cortex in stroke patients. In comparison to functional magnetic resonance imaging (fMRI), fNIRS offers greater convenience and speed, facilitating the design of flexible task paradigms tailored to experimental needs. The F-wave, a polysynaptic spinal reflex, has been investigated by Wang et al. (21), who found that in stroke patients, prolonged F-wave latency and increased amplitude correlated positively with MAS scores. These findings suggest that the F-wave may serve as an objective and quantifiable neuroelectrophysiological indicator of motor neuron excitability in the anterior horn of the spinal cord.

The current study aims to explore the clinical efficacy of acupuncture in combination with cTBS for the treatment of PSS in the upper limbs. Furthermore, we intend to utilize fNIRS and F-wave assessments to delve into the changes in cortical and spinal excitability in PSS patients before and after treatment, aiming to provide preliminary mechanistic insights.

## 2 Methods

### 2.1 Patients

Stroke patients were recruited from the rehabilitation center of the First Affiliated Hospital of Tianjin University of Traditional Chinese Medicine between November 16, 2023 and March 31, 2024. All participants were informed of the study protocol and signed an informed consent form. The study was approved by the Ethical Review Board of the First Affiliated Hospital of Tianjin University of Traditional Chinese Medicine (Ethics No. TYLL2023[Z] 046). Meanwhile, the study has been successfully registered on the website of Chinese Clinical Trial Registry, with registration number ChiCTR2400083631.

The inclusion criteria were as follows: (1) patients with a confirmed first stroke by MRI or CT; (2) unilateral hemiparesis; (3) stable condition; (4) Brunnstrom’s stage III–IV; (5) MAS score >1; (6) age between 40–70 years old; (7) Minimum Mental State Examination (MMSE) score >23; and (8) good adherence to interventions. The exclusion criteria were as follows: (1) brainstem or cerebellar stroke; (2) history of an epileptic seizure, skull fracture, or skull defect; (3) metal or electronic device implanted in the body; (4) pregnant or breastfeeding women; (5) severe damage to organs such as the heart, lungs, liver, kidneys, etc., which could not tolerate the training; and (6) patients who were unable to communicate, such as those who had motor aphasia or mixed aphasia.

### 2.2 Experimental design and procedure

**Figure 1:**
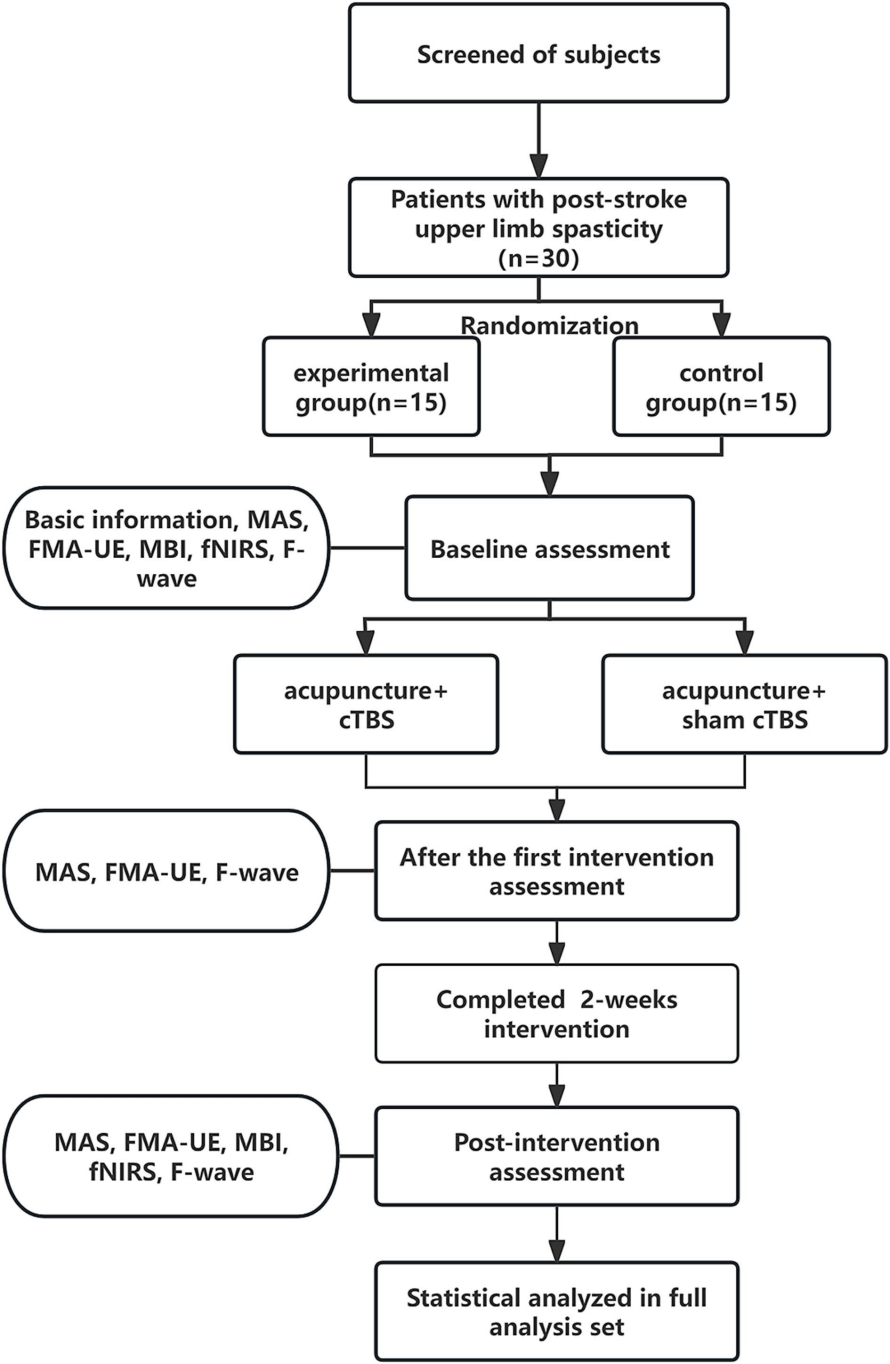
Schematic diagram of the trial process. MAS, Modified Ashworth Scale; FMA-UE, Fugl-Meyer Assessment-Upper Extremity; MBI, Modified Barthel Index; fNIRS, functional near-infrared spectroscopy.

A prospective, single-blind, randomized controlled study was designed, assigning patients randomly to experimental group or control group, ensuring blindness to group allocation. Drawing upon the findings of a prior study (22), we set the two-sided significance level (*α*) at 0.05 and the power of the test (1 − *β*) at 0.9. Furthermore, we ensured that the ratio of sample sizes between the experimental and control groups remained 1:1. Utilizing the methodology proposed by Ye and Yi (23), we employed the R programming language to calculate the necessary sample size, yielding 11 cases for each of the experimental and control groups. Taking into account a potential 20% loss due to missed visits and refusals, we rounded up the required sample size to a minimum of 14 cases per group, resulting in a total sample size of 30 cases (rounded to the nearest whole number for practical purposes). Random allocation codes, generated using SAS 9.4 by an independent third party, sealed in envelopes, determined group assignment. Researchers sequentially enrolled subjects, unaware of their allocation, who then underwent daily cTBS or sham cTBS followed by a 30-min acupuncture session each day for 2 weeks, totaling 10 interventions. Evaluations were conducted at baseline, post-first treatment, and after 2 weeks of treatment. A blinded and trained researcher assessed outcomes using the Modified Ashworth Scale (MAS), the Fugl-Meyer Assessment for Upper Extremity (FMA-UE), the Modified Barthel Index (MBI), fNIRS, and F-wave analysis. The study protocol is depicted in Figure 1.

### 2.3 Therapeutic method

All patients underwent medication therapy tailored to address their underlying conditions and participated in conventional rehabilitation training (24). The rehabilitation protocol entailed positioning the affected limb in a functional posture, facilitating large joint mobilization, and performing passive stretching exercises to alleviate muscular spasticity. On this basis, the experimental group underwent “Regulating Yin and Yang” acupuncture treatment in combination with a real cTBS protocol, whereas the control group received the same acupuncture treatment but in conjunction with a sham cTBS protocol. Each participant received one treatment session daily for 2 weeks, totaling 10 treatment sessions.

#### 2.3.1 “Regulating Yin and Yang” acupuncture program

All patients were positioned supine and underwent sterilization at the designated sites with single-use, sterile Huatuo acupuncture needles of varying sizes (0.25 × 40 mm and 0.30 × 75 mm). The primary focus of acupuncture was on the upper extremities, performed consistently by a qualified acupuncturist. Key acupuncture points identified included Jianyu (LI 15), Quchi (LI 11), Waiguan (TE 5), Shousanli (LI 10), Hegu (LI 4), and Houxi (SI 3). Following the attainment of “de qi,” needles were retained for 30 min. For Qingling (HT 2) and Bizhong (EX-UE), rapid needling techniques were applied without needle retention. Localization of acupoints adhered to the standardized guidelines outlined in “Acupoint Names and Localization” (GB/T 12346-2006) (25), with detailed point placement and procedural steps summarized in Table 1.

**Table 1:** “Regulating Yin and Yang” acupuncture method.

Acupoints	Operating method	Selection basis
Jianyu (LI15)	The needle was penetrated from Jianyu to Binao (LI14), with a needle entry depth of 50–60 mm	All three are the acupoints on the Large Intestine Channel of Hand Yangming, and Quchi is its He-Sea acupoint. Needling the Hand Yangming Channel can enhance the yang qi of the limbs on the affected side of hemiplegia, thereby balancing the yin and yang on the hemiplegic side
Quchi (LI11)	The acupuncture needle was penetrated from Quchi to Shaohai (HT3), with a needle entry depth of 40–50 mm	
Shousanli (LI10)	The acupuncture needle was penetrated 30–35 mm vertically	
Waiguan (TE5)	The acupuncture needle was penetrated from Waiguan to Neiguan (PC6), with a needle entry depth of 40–50 mm, while reinforcing manipulation of twirling and rotating	Waiguan connects with Yang Heel Vessel and Neiguan communicates with Yin Heel Vessel, both of which belong to the Eight Confluent acupoints, and the two acupoints penetrate each other to regulate the operation of the meridian qi in the surface and in the interior
Hegu (LI4)	The acupuncture needle was penetrated from Hegu to Houxi, with a needle entry depth of 50–60 mm. The doctor slightly shook the handle of the needle using the reinforcing method of lifting and thrusting until the palm actively cooperated with the extension	Hugu is the Yuan-Source acupoint and Houxi is one of the Eight Confluent acupoints, which is related to Governor Vessel. Penetrating from Hegu to Houxi can stimulate the deep branch of the ulnar nerve and the branch of the median nerve, and is the main treatment for constrictive pain in the elbow, arm and little finger
Houxi (SI3)		
Qingling (HT2)	The patient flexed the elbow at an internal angle of 120°. The doctor held the wrist joint of the affected limb with his hand, then the needle was penetrated 30–35 mm vertically. Using the reducing method of lifting and thrusting, the patient performed external rotation pumping thrice using the hand. Then the needle was removed	Qinglin is the acupoint on the Heart Channel of Hand-Shaoyin, characterized by a relatively small amount of blood and abundant qi. It can nourish the meridian qi, moisten the essence, and nurture the spirit of the heart
Bizhong (EX-UE)		By needling Bizhong, stimulation can reach the underlying radial nerve trunk, inducing reorganization of local tissue nerve fibers around the acupoint

#### 2.3.2 cTBS and sham cTBS paradigm

**Figure 2:**
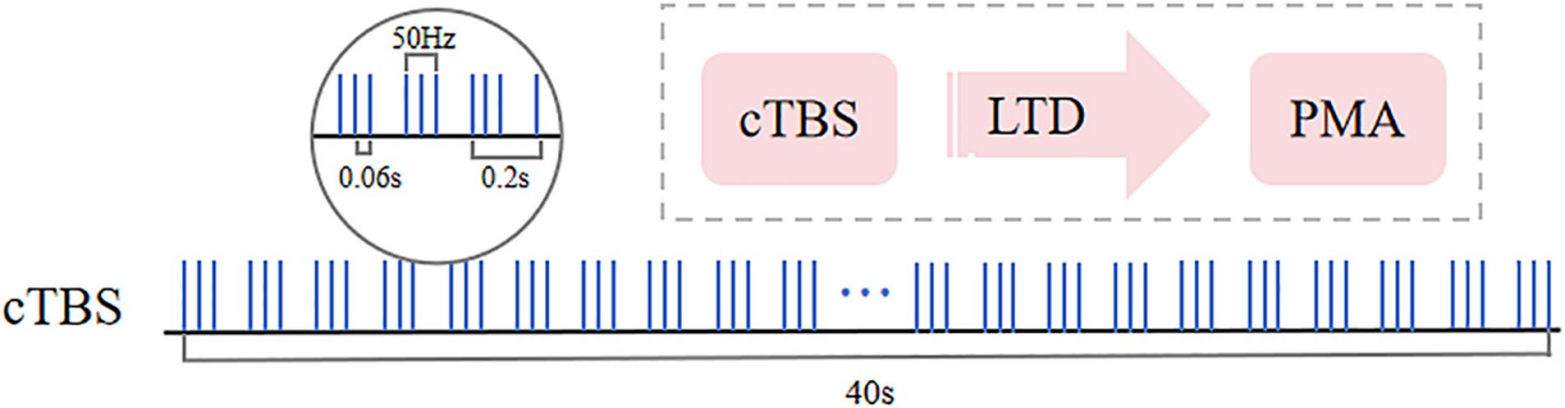
Continuous theta burst stimulation (cTBS) paradigm and neurophysiological effects. The fundamental element of cTBS comprises a burst of 3 stimuli delivered at 50 Hz (each lasting 60 ms), repeated every 200 ms, resulting in an uninterrupted 40-s TBS sequence (totaling 600 pulses). Following a 10-min rest period, the stimulation is repeated with an additional 600 pulses.

The motor threshold (MT) was determined beforehand, and patients were then stimulated with a figure-8 coil of an rTMS stimulator (YRD CCY-I, Wuhan). In a relaxed sitting position, the stimulation targeted the area eliciting maximum motor evoked potential (MEP) in the contralateral first dorsal interosseous (FDI) muscle. MT was defined as the minimum intensity required to induce MEPs of ≥50 μV in at least 5 of 10 trials, specifically when stimulating for thumb abduction (26). Subjects then lay supine and relaxed while the therapist positioned the coil over the healthy PMA, the dorsal portion of the superior precentral sulcus (above the superior frontal sulcus) in the contralateral hemisphere. The cTBS parameters were designed based on the theta-burst stimulation paradigm proposed by Huang et al. (27), which induces long-term depression (LTD) to specifically suppress hyperexcitability in the contralesional motor cortex, thereby ameliorating interhemispheric inhibition imbalance (28). The stimulation intensity was set at 80% of the active motor threshold (AMT) to balance safety and neuromodulatory efficacy (29). Each subject received 40-s cTBS on the contralateral PMA at 50 Hz, with 3 pulses per cluster, 0.06 s per pulse, and intervals of 0.2 s (i.e., 5 Hz), totaling 600 pulses. The stimulation protocol was then repeated after a 10-min rest period. Figure 2 illustrates the cTBS stimulation paradigm used in the study.

The sham stimulation mirrored the intervention parameters and stimulation site of cTBS, however, it involved a 90-degree rotation of the coil. This manipulation ensured that while the patient experienced the sensation of being stimulated, the stimulation itself did not penetrate to the deep surface of the skull.

### 2.4 Assessment method

To ensure rigor and objectivity, a blinded investigator systematically assessed all indicators at predefined time points: pre-treatment, immediately post-treatment, and 2 weeks ± 2 days post-completion.

#### 2.4.1 Scale assessment

Primary outcome was the MAS, a validated tool adhering to the “one-second rule” for precise spasticity severity scoring (0–4+) (30). Secondary outcomes included FMA-UE (max 66) and MBI (100 = full independence), comprehensively evaluating motor function and the activities of daily living (31, 32).

#### 2.4.2 fNIRS measurement and data processing

**Figure 3:**
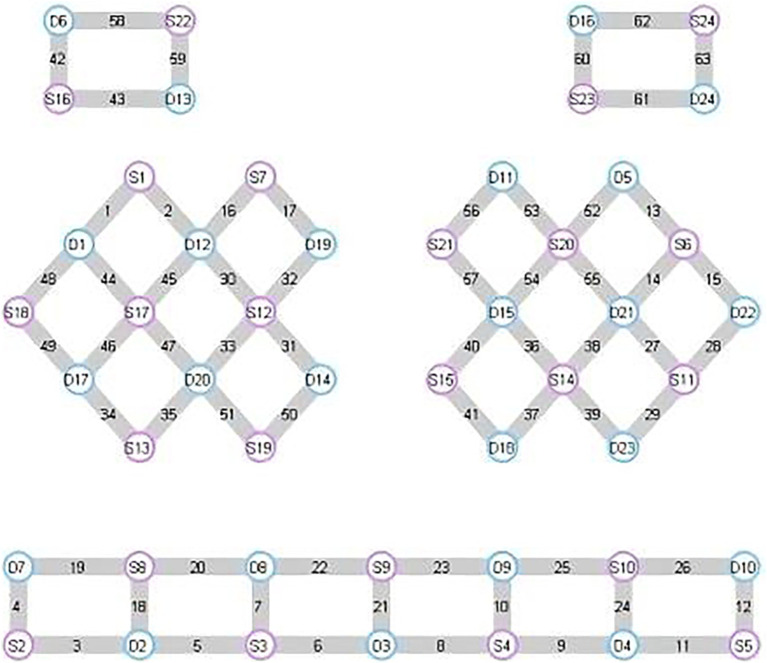
Distribution of light sources, detectors, and channels.

**Figure 4:**
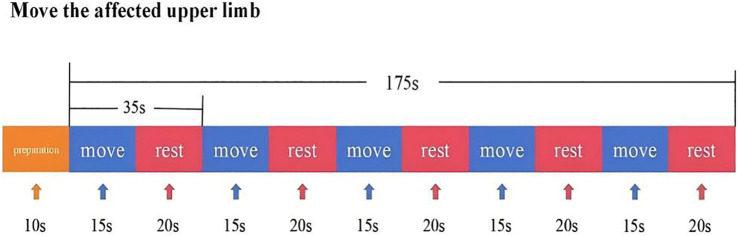
fNIRS task-state design paradigm.

In this experiment, NirScan-6000B equipment (Danyang Huichuang Medical Equipment Co., Ltd., China) was used to continuously measure and record the concentration changes of brain oxygenated hemoglobin (HbO_2_) and deoxyhemoglobin (HbR) during the task. The system consists of a near-infrared light source (light emitting diodes, LED) and an avalanche photodiodes (APD) as detectors, with wavelengths of 730 nm, 808 nm and 850 nm, respectively, and a sampling rate of 11 Hz. The experiment uses 24 light sources and 24 detectors to form 63 effective channels (Figure 3), the average distance between the source and the detector is 3 cm (range2.7–3.3 cm), with reference to the international 10/20 system for positioning. During the resting state, the subject remained sedentary for 8 min. For the task paradigm, the subject was instructed to move the upper limb on the affected side. The subjects rested for 10 s in a quiet state, followed by a 15-s movement of the upper limb on the affected side. Afterward, they rested for 20 s. This task paradigm was repeated for a total of five groups. The task paradigm is illustrated in Figure 4. The NirSpark software (Danyang Huichuang Medical Equipment Co., Ltd., China) package was used to preprocess fNIRS signals and analyze data, it has been used in previous experiments (33). In this study, the frontopolar area (Fp), supplementary motor area & premotor area (SMA and PMA), primary motor cortex (M1), and primary somatosensory cortex (S1) were, respectively, regarded as eight different independent homologous motor networks, namely the regions of interest (ROIs). The functional connectivity of heterologous motor networks was calculated to investigate the strength of functional connectivity (FC) among different ROIs. The settings of ROIs and specific channels are presented in Table 2.

**Table 2:** Settings of region of interest (ROI) and corresponding channels.

ROI of cortical area	Channels	
	Left	Right
Frontopolar area (Fp)	25, 23, 8, 10, 9, 24, 26, 12, 11	22, 6, 7, 20, 5, 18, 19, 4, 3
Pre-motor and supplementary motor cortex (PMA & SMA)	54, 57	30, 32
Primary motor cortex (M1)	15, 14, 56	17, 44, 48
Primary somatosensory cortex (S1)	40, 36, 38, 27	31, 33, 46, 47

#### 2.4.3 F-wave detection and parameter selection

**Figure 5:**
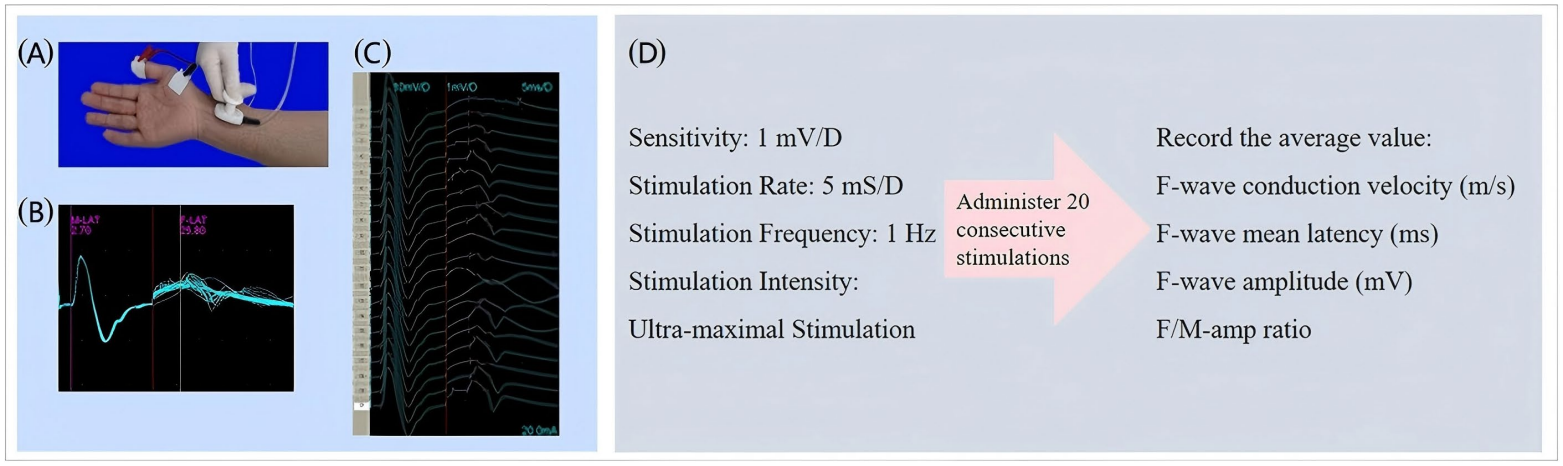
Schematic diagram of F-wave detection. **(a)** Detection position schematic. Stimulation electrode: 1 cm proximal to wrist crease. Recording electrode: On abductor pollicis brevis belly. Reference electrode: On corresponding tendon. Grounding electrode: Attached to dorsal hand. **(b)** F-wave waveform diagram. **(c)** F-wave waveform after 20 superimposed stimulations. **(d)** Relevant parameters for F-wave assessment.

Additionally, F-wave electrophysiology, using the NeuroCare-D1 system, was employed to provide an objective, quantitative assessment of spasticity through standardized procedures and 20 stimulations, enhancing the accuracy of our evaluation (34, 35). Figure 5 shows the schematic diagram of F-wave detection.

#### 2.4.4 Assessment of adverse events

During the trial, all analysis of adverse events (AEs) including bleeding, hematomas, pain, and seizures were closely monitored for their potential association with the trial methodology. Detailed records were kept of AE symptoms, severity, onset, duration, management, and progression. In the event of serious AEs, investigators promptly implemented safety measures and reported the incidents to the subject study unit and ethics committee.

### 2.5 Statistical analysis

To validate our findings, rigorous statistical analysis was performed. The Shapiro–Wilk (S–W) test verified data normality, with continuous variables reported as mean ± SD (x̄ ± s) for normal distributions and median (IQR) for non-normal distributions. Categorical variables were analyzed by chi-square tests and presented as case numbers (ratios) [*n* (%)]. Group comparisons were approach-specific based on data distribution, utilizing the independent t-test for normal data and Mann–Whitney *U*-test for non-parametric data. For repeated measures fitting normal or near-normal distributions, two-way analysis of variance (ANOVA) was employed, considering treatment and time as independent variables. Our main hypothesis targeted the group-by-time interaction. Sphericity violations were adjusted using Greenhouse–Geisser correction, and Bonferroni-corrected *post hoc* tests identified significant group differences. Within-group changes were analyzed by paired t-tests. Multiple comparisons were corrected using the false discovery rate (FDR) (*p* < 0.05), that is, all results that rejected the null hypothesis were corrected for false positives. Statistical significance was set at *p* < 0.05, and all analyses were conducted using IBM SPSS Statistics 25, ensuring reliability and reproducibility.

## 3 Results

### 3.1 Baseline characteristics

This study included 30 patients with upper limb spasms after a stroke, of which males were predominant, accounting for 80% of the entire cohort. Meanwhile, patients with cerebral infarction outnumbered those with cerebral hemorrhage in the disease diagnosis, with a ratio of 5:1. The mean age of the patients was 58.97 ± 2.14 years, and the mean duration of a stroke was 6.5 months. The demographic characteristics and initial clinical assessment parameters of all subjects were evenly distributed between groups and were comparable (*p* > 0.05) (Table 3).

**Table 3:** Baseline characteristics of participants.

Variable		Experimental group (*N* = 15)	Control group (*N* = 15)	*p-*value
Sex, *n* (%)	Male	11 (73)	13 (87)	0.651
	Female	4 (26.67)	2 (13)	
Diagnoses, *n* (%)	Cerebral infarction	14 (93)	11 (73)	0.330
	Cerebral hemorrhage	1 (6.67)	4 (27)	
Hemiplegic side, *n* (%)	Left	10 (67)	6 (40)	0.272
	Right	5 (33)	9 (60)	
Age, year (x̄ ± s)		57.6 ± 2.70	60.33 ± 3.37	0.532
Duration of symptoms, months [M (IQR)]		7 (14)	3 (10)	0.250
MAS-elbow flexor (x̄ ± s)		2.03 ± 0.44	2.00 ± 0.60	0.863
MAS-finger flexor (x̄ ± s)		1.8 ± 0.59	1.60 ± 0.57	0.355
FMA-UE (x̄ ± s)		21.47 ± 11.41	21.87 ± 11.35	0.924
MBI (x̄ ± s)		58.13 ± 17.11	57.40 ± 21.10	0.917

MAS, Modified Ashworth Scale; FMA-UE, Fugl-Meyer Assessment for Upper Extremity; MBI, Modified Barthel Index.

### 3.2 Therapeutic outcomes

#### 3.2.1 MAS

##### 3.2.1.1 MAS-elbow flexor

**Figure 6:**
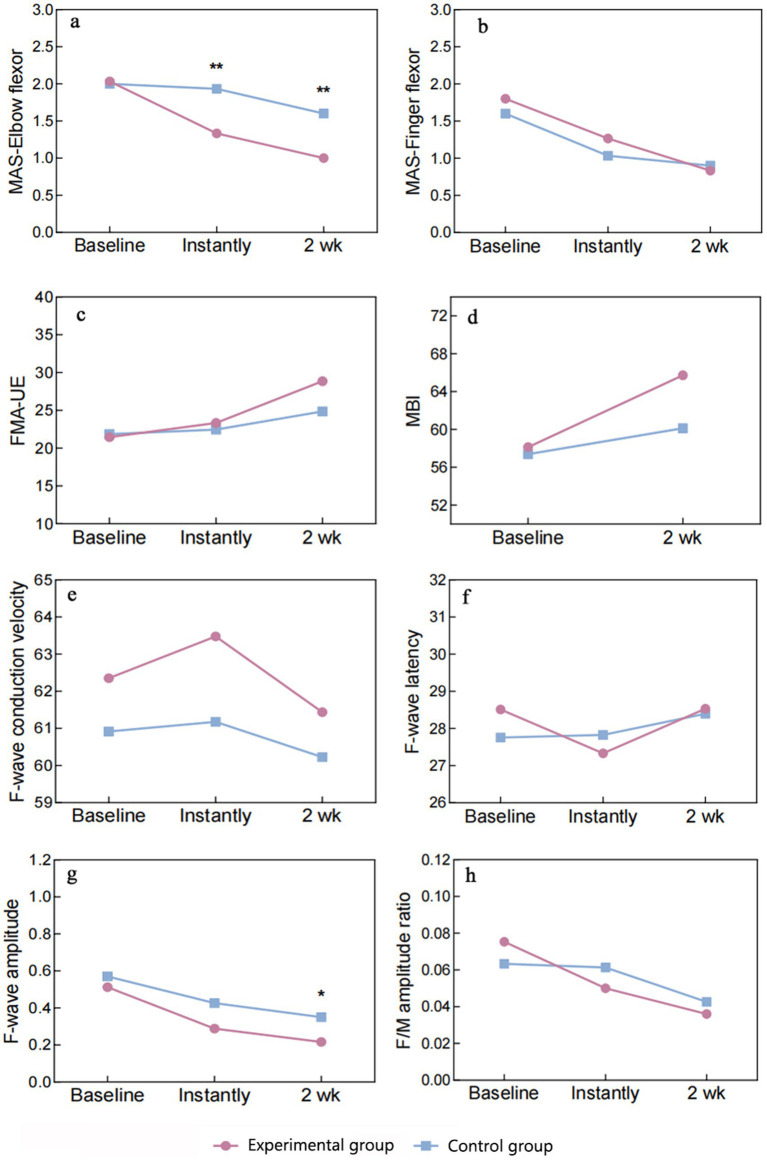
Graph of outcome indicators. MAS, Modified Ashworth Scale; FMA-UE, Fugl-Meyer Assessment-Upper Extremity; MBI, Modified Barthel Index. Significance levels between-group comparisons: **P < 0.01, *P < 0.05.

Data normality and sphericity (Mauchly’s *W* = 0.968, *p* = 0.645) were confirmed, allowing for univariate ANOVA. Repeated measures ANOVA revealed significant effects of groups (*F* = 5.664, *p* = 0.024), MAS-elbow flexor (*F* = 57.194, *p* < 0.001), and their interaction (*F* = 14.865, *p* < 0.001). *Post-hoc* simple effects tests indicated no group difference pre-intervention (*p* = 0.863), but cTBS significantly reduced MAS-elbow flexor compared to sham cTBS post-treatment (*p* < 0.01), persisting at 2 weeks. Within experimental group, MAS-elbow flexor declined progressively over time (*p* < 0.001). In the control group, a statistically significant reduction was solely observed at the 2-week time point (*p* ≤ 0.002) when compared to both baseline and immediately post-treatment measurements. For a detailed breakdown, please refer to Table 4 and Figure 6a.

**Table 4:** Primary and secondary outcomes for the cTBS and sham cTBS groups.

Outcome	Experimental group^a^	Control group^a^	Mean difference^b^	*p*-value^c^
Primary outcome				
MAS-elbow flexor				
Baseline^d^	2.03 ± 0.44	2.00 ± 0.60	0.03 (−0.36, 0.43)	0.863
Instantly^e^	1.33 ± 0.36	1.93 ± 0.53	−0.60 (−0.94, −0.26)	0.001
Mean change from baseline^f^	−0.70 ± 0.46^***^	−0.07 ± 0.26		
2 weeks^g^	1.00 ± 0.46	1.60 ± 0.54	−0.60 (−0.98, −0.22)	0.003
Mean change from baseline^h^	−1.03 ± 0.44^***^	−0.40 ± 0.33^**^		
Mean change from instantly^i^	−0.33 ± 0.36^**^	−0.33 ± 0.30^**^		
MAS-finger flexor				
Baseline	1.80 ± 0.59	1.60 ± 0.57	0.20 (−0.24, 0.63)	0.355
Instantly	1.27 ± 0.72	1.03 ± 0.83	0.23 (−0.35, 0.81)	0.421
Mean change from baseline	−0.53 ± 0.35^***^	−0.57 ± 0.37^***^		
2 weeks	0.83 ± 0.88	0.90 ± 0.82	−0.07 (−0.70, 0.57)	0.832
Mean change from baseline	−0.97 ± 0.52^***^	−0.70 ± 0.49^***^		
Mean change from instantly	−0.43 ± 0.50^**^	−0.13 ± 0.30		
Secondary outcomes				
FMA-UE				
Baseline	21.47 ± 11.41	21.87 ± 11.35	−0.4 (−8.914, 8.114)	0.924
Instantly	23.33 ± 11.64	22.47 ± 11.04	0.867 (−7.616, 9.349)	0.836
Mean change from baseline	1.87 ± 1.36^***^	0.60 ± 1.40		
2 weeks	28.87 ± 12.08	24.87 ± 11.42	4 (−4.791, 12.791)	0.359
Mean change from baseline	7.40 ± 2.44^***^	3.00 ± 1.56^***^		
Mean change from instantly	5.53 ± 2.50^***^	2.40 ± 0.99^***^		
MBI				
Baseline	58.13 ± 17.11	57.40 ± 21.10	0.73 (−13.64, 15.10)	0.917
2 weeks	65.73 ± 18.34	60.13 ± 21.59	5.6 (−9.39, 20.59)	0.450
Mean change from baseline	7.60 ± 5.73^***^	2.73 ± 3.31^**^		
Neurophysiologic indicators-F-wave				
F-wave conduction velocity				
Baseline	62.35 ± 4.22	60.92 ± 4.05	1.436 (−1.656, 4.528)	0.350
Instantly	63.48 ± 4.23	61.18 ± 4.22	2.298 (−0.864, 5.460)	0.148
Mean change from baseline	1.12 ± 3.26	0.26 ± 3.66		
2 weeks	61.44 ± 3.18	60.23 ± 4.49	1.207 (−1.703, 4.116)	0.403
Mean change from baseline	−0.92 ± 5.24	−0.69 ± 5.51		
Mean change from instantly	−2.04 ± 4.98	−0.95 ± 5.64		
F-wave latency				
Baseline	28.51 ± 2.38	27.76 ± 2.34	0.757 (−1.008, 2.523)	0.387
Instantly	27.33 ± 2.64	27.83 ± 2.43	−0.495 (−2.391, 1.401)	0.597
Mean change from baseline	−1.18 ± 1.72	0.07 ± 1.32		
2 weeks	28.53 ± 2.43	28.40 ± 3.24	0.131 (−2.011, 2.272)	0.901
Mean change from baseline	0.02 ± 2.05	0.64 ± 1.24		
Mean change from instantly	1.20 ± 2.19	0.57 ± 1.61		
F-wave amplitude				
Baseline	0.51 ± 0.21	0.57 ± 0.41	−0.058 (−0.301, 0.185)	0.629
Instantly	0.30 ± 0.10	0.43 ± 0.27	−0.138 (−0.293, 0.017)	0.079
Mean change from baseline	−0.22 ± 0.15^***^	−0.14 ± 0.19^*^		
2 weeks	0.22 ± 0.08	0.357 ± 0.20	−0.135 (−0.252, −0.018)	0.026
Mean change from baseline	−0.30 ± 0.21^***^	−0.22 ± 0.25^**^		
Mean change from instantly	−0.07 ± 0.09^*^	−0.08 ± 0.10^*^		
F/M amplitude ratio				
Baseline	0.08 ± 0.04	0.07 ± 0.04	0.012 (−0.016, 0.04)	0.383
Instantly	0.05 ± 0.02	0.06 ± 0.04	−0.011 (−0.037, 0.014)	0.365
Mean change from baseline	−0.03 ± 0.04^*^	−0.002 ± 0.03		
2 weeks	0.04 ± 0.015	0.04 ± 0.03	−0.007 (−0.023, 0.01)	0.408
Mean change from baseline	−0.04 ± 0.04^**^	−0.02 ± 0.03		
Mean change from instantly	−0.01 ± 0.02	−0.02 ± 0.04		

MAS, Modified Ashworth Scale; FMA-UE, Fugl-Meyer Assessment for Upper Extremity; MBI, Modified Barthel Index. ^***^p < 0.001, ^**^p < 0.01, and ^*^p < 0.05 for within-group comparison.

a Values are mean ± SD unless otherwise indicated.

b Calculated as experimental group minus control group. Values are mean (95% confidence interval).

c Between-group post hoc comparison (Bonferroni) unless otherwise indicated.

d Before the first intervention. Values are mean ± SD.

e After the first intervention. Values are mean ± SD.

f Calculated as instantly minus baseline. Values are mean ± SD.

g After 2 weeks of intervention. Values are mean ± SD.

h Calculated as 2-week minus baseline. Values are mean ± SD.

i Calculated as 2-week minus instantly. Values are mean ± SD.

##### 3.2.1.2 MAS-finger flexor

The normality of the data was confirmed by S–W, skewness, and kurtosis tests. Mauchly’s *W* (0.840, *p* = 0.095) indicated adherence to sphericity assumptions, justifying the use of univariate ANOVA. Repeated measures ANOVA revealed non-significant group differences (*F* = 0.224, *p* = 0.639, *η*^2^ = 0.008), but significant effects of MAS-finger flexor (*F* = 58.504, *p* < 0.001, *η*^2^ = 0.676), with no significant interaction (*F* = 2.203, *p* = 0.120, *η*^2^ = 0.073). Multiple comparisons failed to show significant differences in MAS-finger flexor between groups at any time point (*p* > 0.05). In the experimental group, MAS-finger flexor declined progressively over time, with significant paired comparisons (*p* < 0.01). In the control group, significant reductions were observed from baseline to immediate post-treatment and 2 weeks post-treatment (*p* < 0.001), but the difference between these two time points was non-significant (*p* = 0.649). The experimental group exhibited a consistent decrease in MAS-finger flexor, while the control group showed a significant initial drop, with no further significant change at 2 weeks (Table 4 and Figure 6b).

#### 3.2.2 FMA-UE

The S–W test confirmed normal distribution of data. Sphericity was assumed based on Mauchly’s *W* (0.819, *p* = 0.068), allowing univariate ANOVA. Repeated measures ANOVA found non-significant group effects (*F* = 0.127, *p* = 0.724, *η*^2^ = 0.83), but significant FMA-UE effects (*F* = 136.661, *p* < 0.001, *η*^2^ = 0.70) with significant interaction (*F* = 23.742, *p* < 0.001, *η*^2^ = 0.459). Simple effects tests were performed. Group effects were non-significant at all time points (*p* > 0.05), with no consistent FMA-UE differences between experimental group and control group. FMA-UE simple effects were significant in both groups (*p* < 0.001), with experimental group showing continuous improvement across time (*p* < 0.001). Control group exhibited significant improvement from baseline to 2 weeks post-treatment (*p* < 0.001), but not between baseline and immediate time (*p* = 0.310) (Table 4 and Figures 6c).

#### 3.2.3 MBI

Between-group comparisons using the two-sample independent *t*-test revealed no significant difference in the MBI between the experimental group and control group at baseline (*p* = 0.917), which was comparable. The MBI of the experimental group was higher than that of the control group at both the immediate time and 2 weeks post-treatment, with no statistically significant difference (*p* > 0.05). Between-group comparisons using paired two-sample t-tests revealed that the MBI exhibited significant changes in experimental group and control group at 2 weeks post-treatment compared with the baseline period (*p* < 0.01) (Table 4 and Figure 6d).

### 3.3 Mechanism outcomes

#### 3.3.1 F-wave

##### 3.3.1.1 Conduction speed

Data normally distributed. Sphericity violated (Mauchly’s *W* = 0.769, *p* = 0.029), applying Greenhouse–Geisser correction. ANOVA revealed no significant effects of groups (*F* = 2.258, *p* = 0.144) or conduction velocity (*F* = 1.459, *p* = 0.243), and no interaction (*F* = 0.215, *p* = 0.761). Multiple comparisons showed no differences in conduction velocity between experimental and control groups at any time point (*p* > 0.05).

##### 3.3.1.2 F-wave latency

Data normally distributed. Sphericity assumed (Mauchly’s *W* = 0.930, *p* = 0.378). ANOVA found no significant group effect (*F* = 0.022, *p* = 0.882) but a significant main effect of latency (*F* = 4.048, *p* = 0.023). No interaction effect (*F* = 1.981, *p* = 0.148). Experimental group showed significant latency reduction from baseline to immediate (*p* = 0.017), with no other significant changes. Control group showed no significant changes.

##### 3.3.1.3 F-wave amplitude

Data normally distributed. Sphericity violated (Mauchly’s *W* = 0.353, *p* < 0.001), applying Greenhouse–Geisser correction. ANOVA revealed a significant main effect of amplitude (*F* = 34.567, *p* < 0.001) but no group or interaction effects. Experimental and control groups differed significantly in amplitude after 2 weeks (*p* = 0.026), with significant intra-group changes over time (*p* < 0.05).

##### 3.3.1.4 F/M-amp ratio

Data normally distributed. Sphericity assumed (Mauchly’s *W* = 0.951, *p* = 0.506). ANOVA showed a significant main effect of ratio (*F* = 11.287, *p* < 0.001) but no group or interaction effects. Experimental group showed significant changes in ratio from baseline to immediate and 2 weeks (*p* < 0.05), with no change between these time points. Control group showed no significant changes (Table 4 and Figure 6 summarize these findings).

#### 3.3.2 fNIRS

##### 3.3.2.1 Cortical activation results

**Figure 7:**
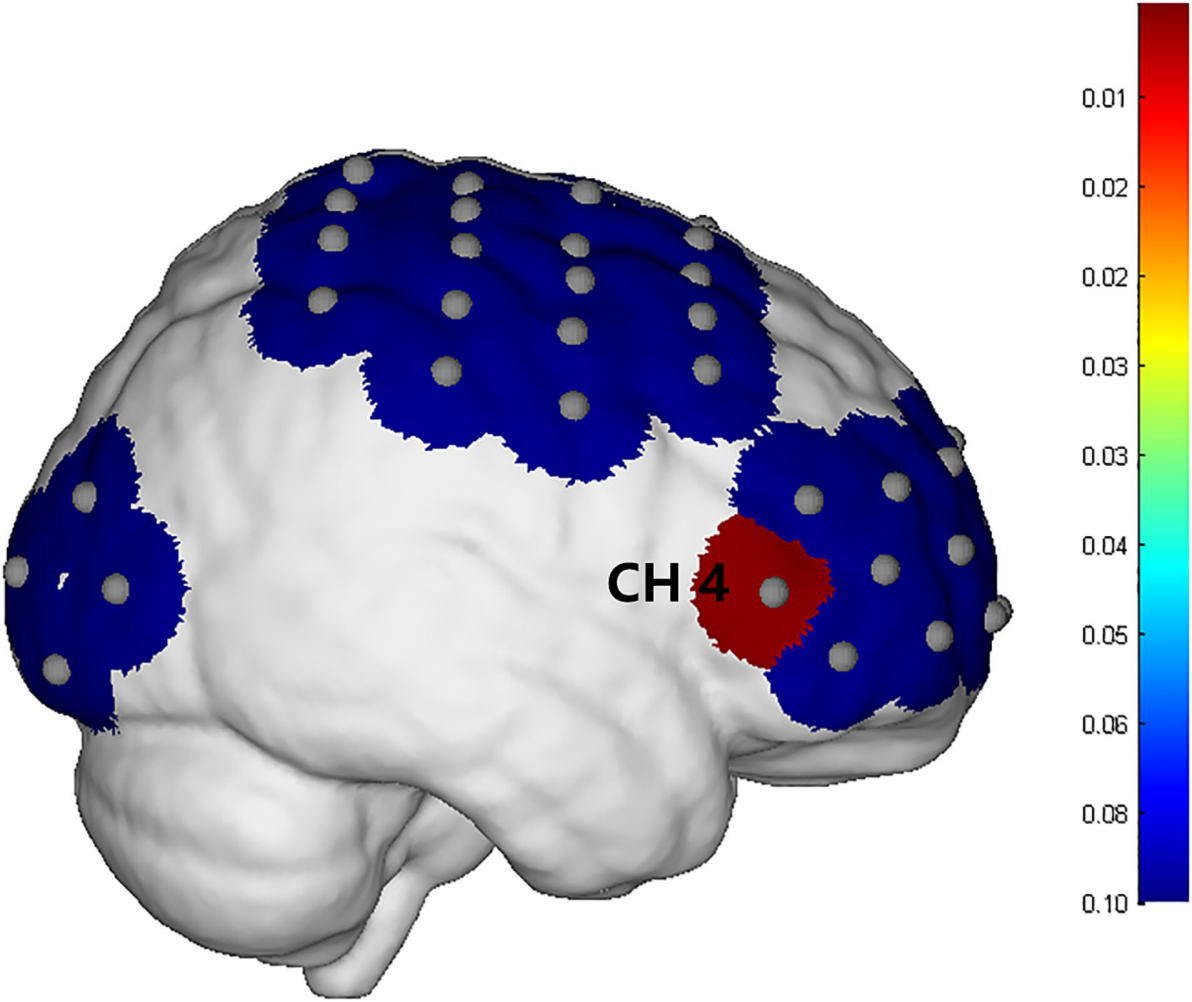
The difference in brain activation between the experimental group and the control group.

The integral value, mean value and difference value of the whole brain HbO_2_ concentration in the experimental group all increased after treatment, but there was no statistical significance (*P* > 0.05). In the control group, the mean, integral, difference and β values of wholebrain HbO_2_ concentration all increased after treatment, but there was no statistical significance (*P* > 0.05). Comparing between groups, there was no significant difference between the experimental group and the control group in the mean, integral, difference and β values before treatment. After treatment, there was no significant difference in the mean, integral and β values between the experimental group and the control group. There was a significant difference (*P* = 0.009) in the difference of channel CH4 (Figure 7), which is located on the right Fp and is arranged in Broca’s area according to the channel arrangement (Table 5).

**Table 5:** The detail information of CH4.

Channel	Light sources and detectors	Brodmann area	Region	MNI coordinates		
				X	Y	Z
4	S2-D7	BA 45	R-pars triangularis Broca’s area	58.608	33.374	5.662

##### 3.3.2.2 Results of brain network connectivity

###### 3.3.2.2.1 Comparison of regions of interest

**Figure 8:**
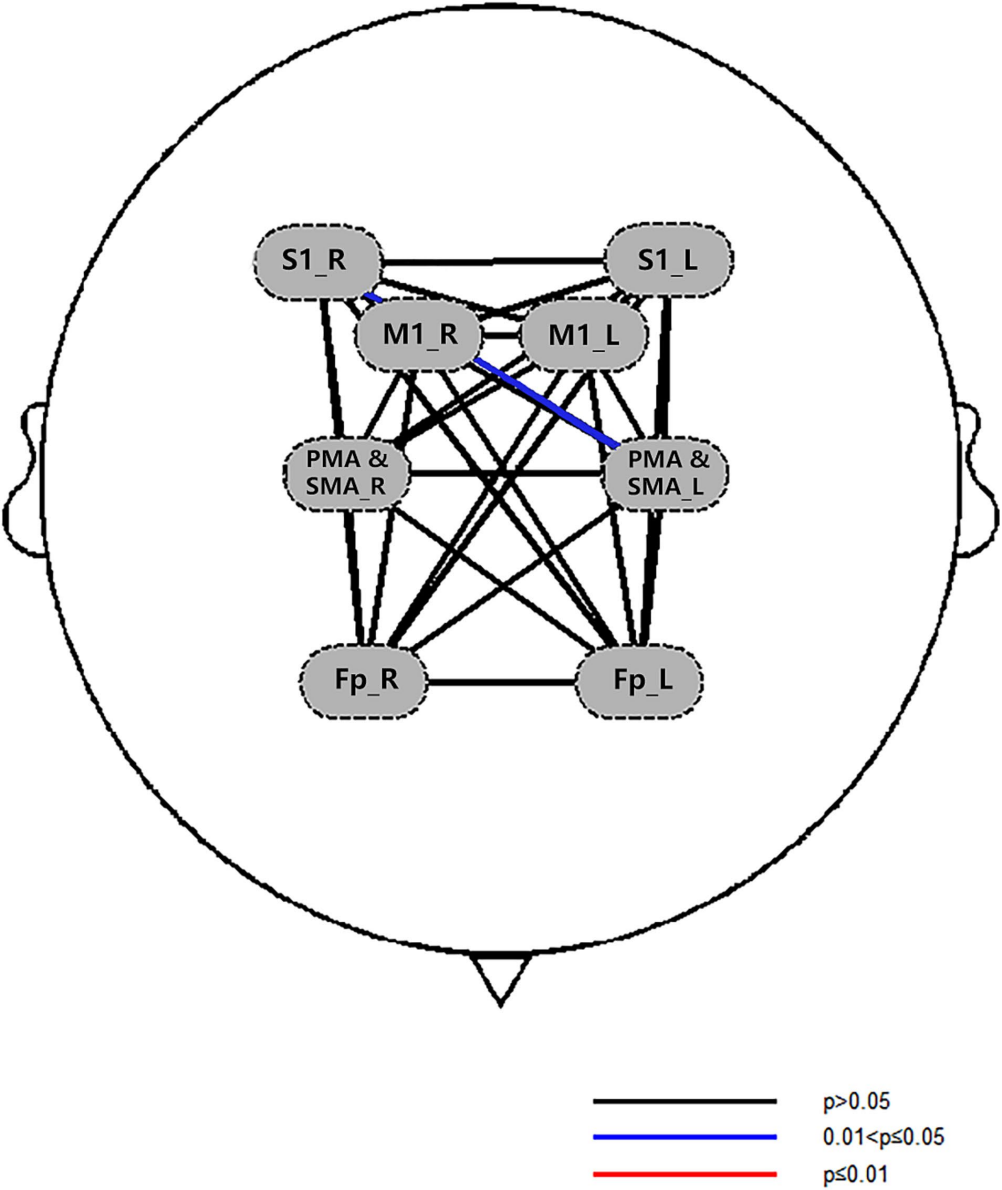
The FC map of experimental group ROI.

After treatment, there were significant differences in FC between the right S1 -the left PMA and SMA in the experimental group (*p* < 0.05), as shown in Figure 8. Left M1-left PMA and SMA (*p* = 0.085), right S1-left S1 (*p* = 0.067), and left M1-right S1 FC (*p* = 0.086) appeared marginally significant. There was no significant difference between the ROI before and after treatment in the control group.

###### 3.3.2.2.2 Comparison among channels

**Figure 9:**
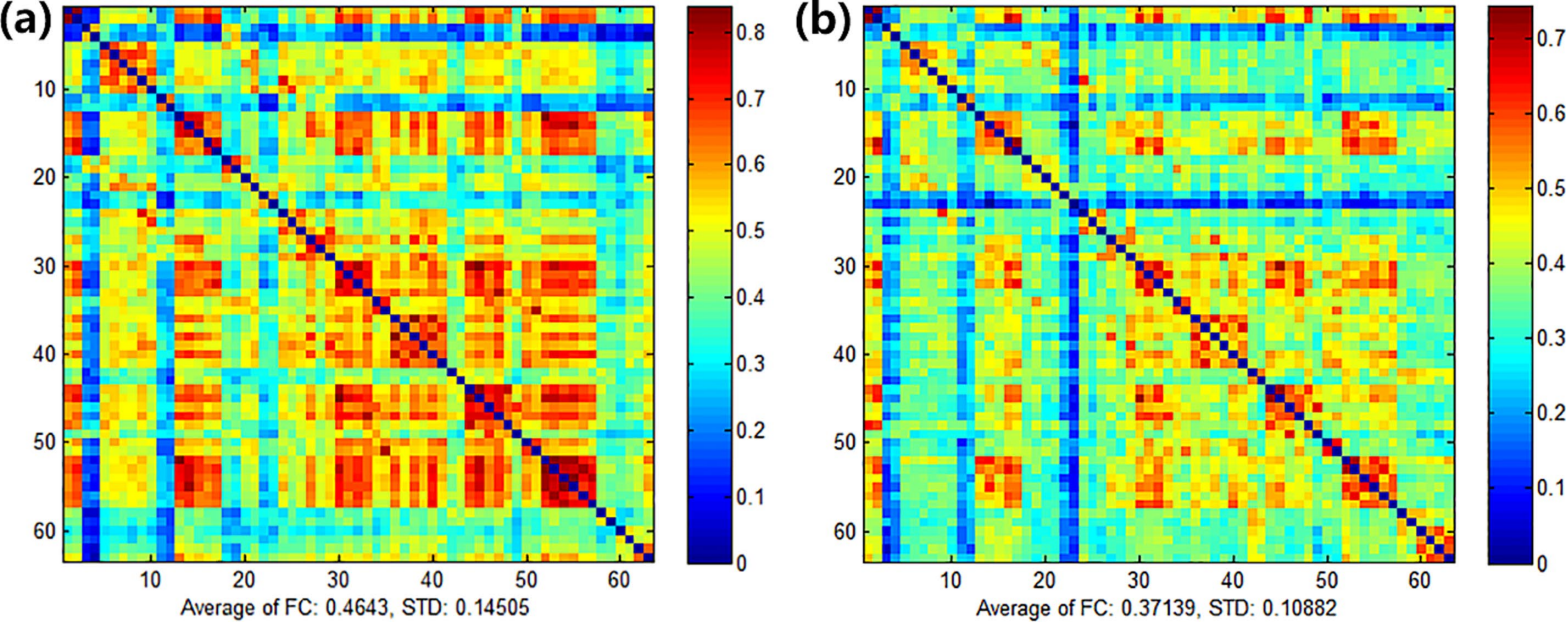
FC of the experimental group **(a)** and the control group **(b)** before treatment.

**Figure 10:**
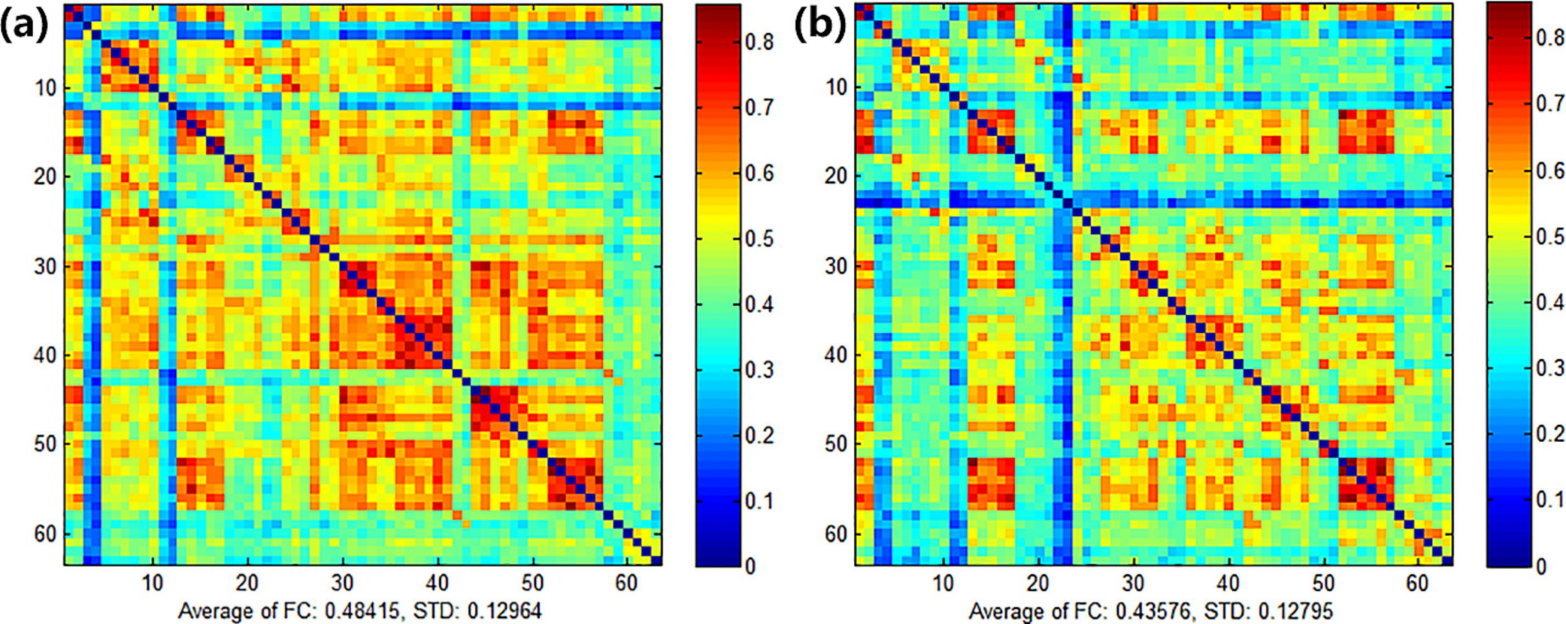
FC of the experimental group **(a)** and the control group **(b)** after treatment.

After treatment, the functional connectivity (FC) in both groups increased compared with that before treatment, yet no significant differences were observed. For the comparison between groups, there were no obvious differences between the experimental group and the control group before treatment (see Figure 9). Also, there were no obvious differences between the experimental group and the control group after treatment (see Figure 10). However, it can be observed from the figures that the FC in the experimental group tended to be higher than that in the control group.

### 3.4 Security analysis

Throughout the trial, only one instance of subcutaneous bleeding was noted in the experimental group following acupuncture. The bleeding was promptly addressed by applying pressure with a sterile cotton ball, and no additional AEs were reported.

## 4 Discussion

Spasticity is a prevalent post-stroke limb movement disorder, significantly impeding the rehabilitation progress of hemiplegic patients. Recent research indicates that approximately half of stroke patients begin to exhibit symptoms of joint stiffness around the 10th day post-stroke. The incidence of spasticity in upper limb joints and the wrist joint increases from day 10 to the third month, remaining constant from month 3 to month 12 (36, 37). Investigating the progression patterns of spasticity contributes to the development of rehabilitation protocols for pre-hemiplegic stages, offering effective measures to mitigate the incidence and severity of spasticity.

Building upon the foundational principles of traditional Chinese medicine and contemporary rehabilitation theories, we invented the “Yin and Yang Regulation” acupuncture technique. According to the Brunnstrom stages of stroke, discernible differences in yin-yang dynamics exist across various stages. Employing channel pattern differentiation for treatment, distinct needling techniques tailored to each stage were applied. Spasticity predominantly manifests in stages III–IV post-stroke as “urgent yin and slow yang.” The needling approach primarily focuses on reducing the yin channels and supplementing the yang channels, with a notable emphasis on the characteristic technique of penetration needling during this stage. The specific rationale behind the selection of acupuncture points is outlined in Table 1. The clinical efficacy of this acupuncture technique has been confirmed by our previous research studies (38, 39). Clinical evidence supports acupuncture’s significant advantage in ameliorating distal limb spasticity, such as hand contracture and foot inversion after a stroke (40, 41).

Innovatively, our study focused on cTBS, a unique TMS mode that induces long-lasting depression-like reductions in MEP and corticospinal activity post-stimulation (42), offering a novel strategy for post-stroke neurological rehabilitation. cTBS applied to healthy hemispheres has been shown to enhance neurophysiological effects, positively influencing motor recovery in subacute stroke patients (29). When combined with early upper limb training, cTBS stimulation of the healthy primary motor cortex accelerated upper limb motor recovery and prognosis (43). Furthermore, cTBS over the right inferior parietal lobe facilitated hand function recovery (44). Notably, cTBS demonstrated improved upper limb spasticity symptoms compared to rTMS, with shorter application durations (16). Given its short stimulation time and pronounced modulation of cortical excitability, cTBS was chosen to optimize therapeutic effects on PSS.

The cTBS parameters adopted in this study (50 Hz intraburst frequency, 3 pulses per burst, 5 Hz interburst frequency, and 600 total pulses) are grounded in theoretical foundations and validated by prior research. Huang et al. (27) demonstrated that the 5 Hz theta rhythm aligns with the brain’s natural oscillatory frequency, thereby enhancing synaptic plasticity. Intraburst high-frequency stimulation (50 Hz) induces LTD in the target cortex by modulating NMDA receptor-dependent synaptic efficacy, effectively reducing contralesional motor cortex excitability (45). Furthermore, the 600-pulse protocol has been validated in multiple randomized controlled trials for stroke rehabilitation. For instance, Suppa et al. (45) reported that 600-pulse cTBS significantly improves upper limb motor function in stroke patients (Fugl-Meyer score increase ≥8 points), with sustained effects up to 3 months post-treatment. Additionally, the interburst interval (200 ms) was designed in accordance with international safety guidelines for non-invasive brain stimulation (46), ensuring focused stimulation energy on the target cortical area while mitigating coil overheating risks. This parameter combination leverages the synergistic effects of “high-frequency intraburst stimulation and low-frequency interburst rhythm” to achieve effective contralesional inhibition while avoiding neuronal overstimulation-induced fatigue (43). Notably, although the current parameters are well-supported by existing literature, future studies should explore individualized parameter optimization (e.g., dynamic adjustment based on resting motor threshold) to further enhance therapeutic outcomes.

The primary motor cortex (M1) region of the brain was used as a stimulation target in previous studies to promote neural function remodeling in the brain and improve limb motor function. However, no significant effect of this stimulation target on MAS score changes was found (39). Therefore, we attempted to identify new stimulation targets to relieve spasticity symptoms in stroke patients. The PMA, situated anteriorly to the M1 area, serves as the cortical hub of the extrapyramidal system, functioning as an advanced cortical center that governs reticulospinal tracts and modulates limb muscle tone (18, 47). Based on the principle of bi-directional hemispheric balance, we applied inhibitory stimuli to the PMA on the healthy side of the stroke patients, thereby attenuating the facilitating effect on the pontine reticular formation, which in turn reduces the hyperexcitability of retrosplenial tracts and results in the alleviation of spasticity symptoms.

We used cTBS combined with “regulating Yin and Yang” acupuncture method to stimulate the PMA of the healthy hemisphere and found that cTBS combined with acupuncture significantly improved upper limb spasticity in stroke patients, especially elbow spasticity, which was significantly better than that of the control group, after the evaluation of the MAS score and the measurement of the F-wave of the median nerve of the affected side at the baseline period, the immediate period, and 2 weeks post-intervention. This suggests that cTBS can significantly enhance the antispasmodic effect of “regulating Yin and Yang” acupuncture and make up for the insufficiency of acupuncture in relieving proximal limb spasms, and the advantages of the combined therapy are manifested in the immediate and short-term period after the intervention.

The minimal clinically important difference (MCID) defines the threshold of beneficial change perceived by patients, excluding adverse effects or costs (48, 49). Chen et al. (50) established MCID values for MAS at 0.48 (moderate) and 0.76 (good). Hsueh et al. (51), using criterion and distribution methods, determined the MCID for FMA-UE in 50 recent stroke survivors to be 1.0–8.4. Page et al. (52), in a study of 146 chronic stroke patients, reported clinically significant FMA-UE changes within 4.25–7.25 points. Chen et al. (53) reported an MCID of 4.6 points for FMA-UE in 56 stroke patients. Huang et al. (54) indicated an MCID of 5.34 points for MBI in ischemic stroke patients, suggesting a clinically meaningful change of ≥6 points, excluding measurement errors. The current trial yielded effect values of 0.77 for MAS change, 7.40 for FMA-UE change, and 7.60 for MBI change after 2 weeks of treatment with cTBS combined with acupuncture, which are all higher than the currently accepted MCID effect values. This indicates that acupuncture combined with cTBS can improve upper limb spasticity status, upper limb motor function, and daily life ability of patients with upper limb spasticity after a stroke, and there exists an important clinical value.

The cerebral cortex is regarded as the higher center of human life activities (55). For patients with upper limb spasticity after stroke, the functional state of their cerebral cortex has a decisive influence on the rehabilitation process and prognosis. As an emerging brain imaging method, fNIRS has become an important tool for evaluating the functional status of the cerebral cortex (56). Compared with fMRI, fNIRS is more convenient and can flexibly set the task-state activity paradigm according to the specific conditions of the patient and the specific needs of the experimental design. At the same time, this technology can also combine the functional connectivity in the resting state to conduct a comprehensive and in-depth assessment of the brain functional network of patients with spasticity. Therefore, fNIRS can be strongly recommended as a powerful tool to study and understand the mechanism of post-stroke motor function recovery (57). Research using fNIRS shows that physical intervention can effectively promote the activation level of the sensorimotor cortex in patients with stroke limb spasticity, and the activation changes in the contralateral SMA are particularly significant. This discovery not only reveals the remodeling process of cortical function in spastic patients during rehabilitation training, but also verifies the effectiveness and potential of fNIRS technology in exploring the mechanism of post-stroke motor function recovery.

In this study, we observed significant brain activation differences on channel CH4 between the two groups. The location of channel CH4 is the right Broca area. Therefore, the combined therapy of acupuncture and cTBS significantly could improve post-stroke upper limb spasticity by enhancing activation in the right Broca area. This mechanism contrasts with the findings of Mayorova et al. (58), who reported a negative correlation between compensatory activation of the SMA and spasticity severity in patients. Furthermore, Wei et al. (59) identified PMC plasticity as a core mechanism for spasticity improvement. Normally, we would expect that when a motor task is activated, the patient’s motor cortex would show significant changes, rather than Broca’s area. However, through in-depth research on the relevant literature, we found that at least part of Broca’s area overlaps with the ventral premotor cortex (55). In addition to common language functions, Broca’s area also has non-language related motor functions including complex hand movements, associative sensorimotor learning, and sensorimotor integration (60). This shows that Broca’s area is involved in the planning and control of complex movements to a certain extent. In this study, stroke patients were treated with acupuncture combined with cTBS of the ventral PMA, which further improved their ability to execute and control motor commands. We hypothesize that the therapeutic effects of the combined intervention may arise from remodeling of the Broca area-motor circuit, a pathway underexplored in prior studies. Notably, the lack of significant changes in motor cortex activation observed here may stem from insufficient statistical power due to the small sample size or indicate that the therapy preferentially targets higher-order motor planning networks. After treatment, the FC maps of the two groups of patients showed obvious differences. Although the results were at a marginally significant level, considering the small sample size of this study, we can still think that this combination therapy can cause patients to changes in brain functional status, thereby promoting the remodeling process of brain function.

F-wave analysis enhanced understanding of nerve conduction and excitability. No significant disparities in F-wave velocity or latency were noted between groups, possibly attributed to spasticity improvement primarily stemming from cortical modulation, rather than alterations in peripheral nerve conduction. Notably, acupuncture combined with cTBS induced marked variations in F-wave amplitude and F/Mamp ratio, indicative of modulated neural excitability and synaptic plasticity changes. These findings reinforce the neurophysiological rationale for acupuncture combined with cTBS in alleviating upper limb spasticity.

In conclusion, this study introduces novel treatment parameters (cTBS) and a specific stimulation target (PMA) combined with acupuncture for upper limb spasticity after stroke. Our study demonstrates that the combination of acupuncture and cTBS yields relatively satisfactory immediate and short-term improvements in upper limb spasticity among stroke patients, including enhancing upper extremity motor function and facilitating activities of daily living. This approach compensates for the limitations of acupuncture alone in addressing proximal limb spasticity and potentiates its antispasticity effects. At the same time, acupuncture combined with cTBS can affect the brain functional connection pattern, promote cerebral blood oxygen metabolism and cortical activation, especially in Broca’s area, and reduce the excitability of neurons in the spinal cord.

The utilization of cTBS to stimulate the PMA of the contralateral cerebral hemisphere as a means of augmenting the effectiveness of acupuncture represents a promising alternative therapeutic approach, offering potential advantages to both healthcare providers and patients alike. However, as a preliminary study, this research has several limitations: (1) the relatively small sample size may restrict statistical power and generalizability; (2) the short treatment duration and absence of long-term follow-up preclude conclusions on sustained therapeutic effects; and (3) the single-blind design, coupled with potential residual placebo effects from sham cTBS, introduces bias risks. Future investigations should prioritize large-scale, multicenter trials with extended treatment periods and multiple follow-up timepoints. Adopting a double-blind design would rigorously isolate treatment-specific effects from placebo influences. Additionally, integrating patient-reported outcome measures (PROMs) could refine efficacy quantification by capturing subjective improvements and placebo contributions. To comprehensively validate these findings, multimodal approaches—such as combining neurophysiological monitoring (e.g., TMS-EEG), functional neuroimaging (e.g., fMRI), and biomarker analysis—are essential to elucidate the neuroplasticity mechanisms underlying combined therapies and optimize personalized intervention strategies.

## Data Availability

The raw data supporting the conclusions of this article will be made available by the authors, without undue reservation.

## Glossary

cTBS: Continuous theta burst stimulation
MAS: Modified Ashworth Scale
FMA-UE: Fugl-Meyer Assessment-Upper Extremity
MBI: Modified Barthel Index
F/M-amp: F-wave/M-wave amplitude
MD: Mean difference
PSS: Post-stroke spasticity
RST: Reticulospinal tract
TMS: Transcranial magnetic stimulation
rTMS: Repetitive transcranial magnetic stimulation
LTD: Long-term depression
PMA: Premotor area
MMSE: Minimum Mental State Examination
MT: Motor threshold
MEP: Motor evoked potential
FDI: First dorsal interosseous muscle
AMT: Active motor threshold
AEs: Adverse events
ADLs: Activities of daily living
S–W: Shapiro–Wilk
ANOVA: Analysis of variance
M1: Primary motor cortex
MCID: Minimal clinically important difference
Fp: Frontopolar area
PMA and SMA: Pre-motor and supplementary motor cortex
M1: Primary motor cortex
S1: Primary somatosensory cortex
fNIRS: Functional near-infrared spectroscopy
fMRI: Functional magnetic resonance imaging
FDR: False discovery rate
FC: Functional connectivity
PROMs: Patient-reported outcome measures
